# Radiographical evaluation of ulcerative colitis

**DOI:** 10.1093/gastro/gou026

**Published:** 2014-05-19

**Authors:** Parakkal Deepak, David H. Bruining

**Affiliations:** Division of Gastroenterology and Hepatology, Mayo Clinic College of Medicine, Rochester MN, USA

**Keywords:** ulcerative colitis, inflammatory bowel disease, radiographical evaluation

## Abstract

Radiographical modalities have become important diagnostic tools in cases of ulcerative colitis (UC). Imaging can be used non-invasively to determine the extent of involvement, severity of disease and to detect disease-related complications and extra-intestinal inflammatory bowel disease (IBD) manifestations. While abdominal X-rays and barium enemas still retain their relevance in specific clinical settings, the use of computed tomography enterography (CTE) or magnetic resonance enterography (MRE) are now used as first-line investigations to exclude active small bowel disease in IBD patients and can be utilized to detect active colonic inflammation. Additionally, CT colonography and MR colonography are emerging techniques with potential applications in UC. Ultrasonography, leukocyte scintigraphy and positron emission tomography are novel abdominal imaging modalities currently being explored for IBD interrogations. This plethora of radiological imaging options has become a vital component of UC assessments.

## INTRODUCTION

Radiographical evaluations are a key component of diagnostic and management algorithms in patients with inflammatory bowel disease (IBD). Due to its predilection for small bowel involvement, imaging in Crohn’s disease (CD) provides clinicians with objective evaluations of small bowel regions that are inaccessible to standard endoscopic techniques, detects strictures and reveals penetrating disease complications. In contrast, ulcerative colitis (UC) involves the rectum, often extending proximally in a continuous fashion throughout the colon [1]. Radiological testing in UC can be used as an alternative to endoscopic assessments, when tissue acquisition is not needed, providing data on disease activity, extent and severity. There is a wide range of options available to clinicians, with the correct modality often being selected, based on both intrinsic test and patient-specific features. This review will explore the role of radiological imaging in the evaluation and management of UC.

## INDICATIONS

In UC, the use of radiological studies has some overlap with CD indications, as well as indications unique to UC (Table 1). Imaging can assist in the non-invasive determination of the extent and severity of disease and identify complications such as toxic megacolon and intestinal perforation. UC imaging indications are also useful in excluding small bowel disease in IBD unclassified type (IBD-U) patients and assessing for alternate etiologies for a patient’s symptoms, such as extra-intestinal IBD manifestations. Radiographical techniques can be utilized after colectomy to evaluate ileo-anal pouch function and anatomy. Potential applications include predicting the need for colectomy or response to medical therapy and assessing bone health in IBD patients at risk for osteopenia or osteoporosis [2].

**Table 1.** Indications for radiographical evaluation in patients with ulcerative colitis

Exclude small bowel disease in patients with IBD, unclassified type (IBD-U)
Exclude alternate etiologies for symptoms and extra-intestinal IBD manifestations
Determine disease activity, extent and severity
Identify disease complications (toxic megacolon and perforation)
Evaluation of the ileo-anal pouch function and anatomy
Emerging potential indications
Predict the need for colectomy
Evaluate response to therapy
Bone health assessments

## ABDOMINAL X-RAYS

**Figure 1.**
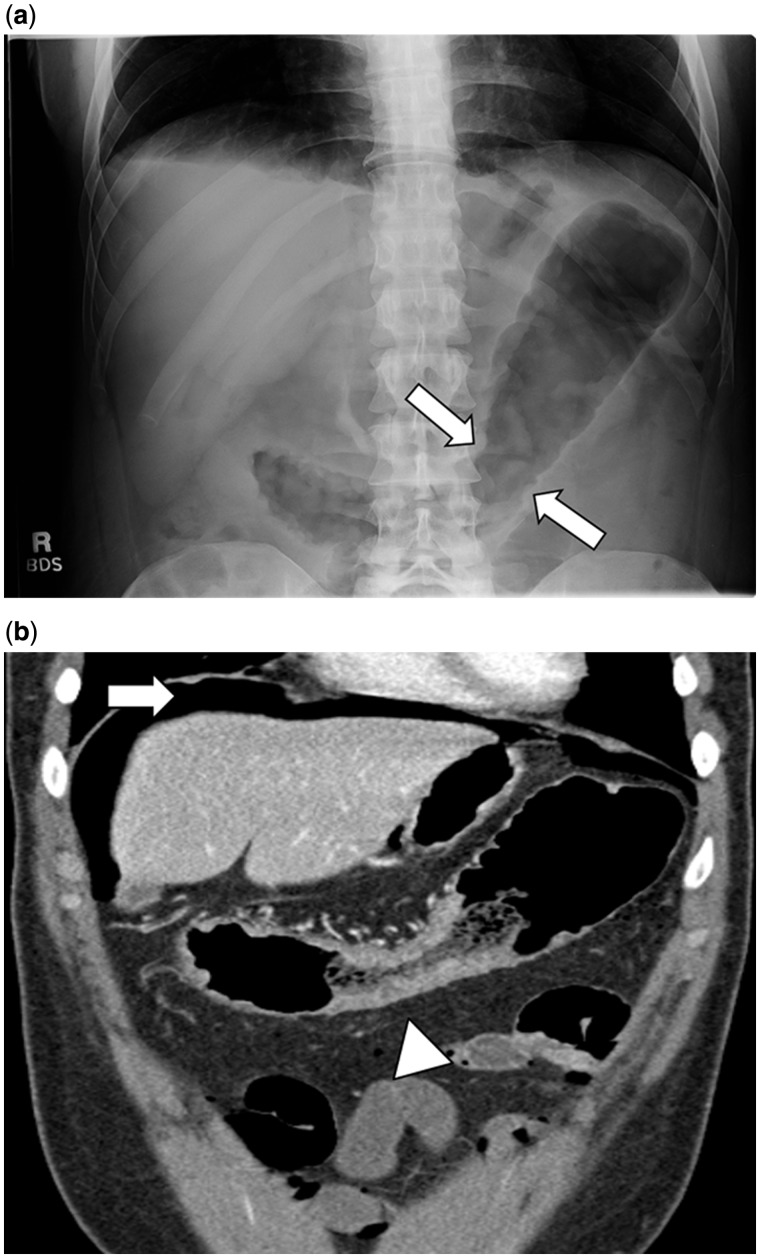
A 32-year-old female patient with ulcerative colitis presenting with abdominal pain and bloody diarrhea. 1a: Abdominal X-ray depicting areas of thumbprinting (white arrow) suggestive of colonic bowel wall edema. 1b: Computed tomography demonstrating free air (white arrow) and colon wall thickening (white arrowhead).

Abdominal X-rays (AXRs) are widely available and can provide crucial information in the acute setting. AXRs are typically performed without the use of intravenous, oral, or rectal contrast. Indications include assessing for perforation of the viscus and toxic megacolon [3, 4]. If perforation is a clinical concern, both supine and upright films should be requested. In patients unable to stand for an upright film, left decubitus positioning is an alternative strategy. Detection of colonic dilatation—defined as total or segmental colonic distension of >6 cm—assists in diagnosing toxic megacolon in the presence of systemic toxicity [4]. Dilatation is typically most evident in the transverse colon, the least-dependent portion of the colon on supine films [5]. Other features of active inflammation include colonic air–fluid levels or loss of colonic haustration [4]. In the presence of severe disease, the colon may exhibit an irregular, nodular contour with mucosal islands separated by severely inflamed mucosa (Figure 1a) [6]. Lastly, AXRs may suggest the extent of disease, as an inflamed colon contains less stool and absence of stool in the colon suggests pancolitis [6].

The performance of AXR has been compared with computed tomography (CT) imaging. The presence of intraperitoneal air is more likely to be missed on abdominal X-ray films and abdominal CT has demonstrated a higher diagnostic yield for detecting IBD-related complications (Figure 1b). In a small study (*n* = 18) by Imbriaco and colleagues, in patients with toxic megacolon (including four individuals with UC), CT imaging detected two perforations that were missed on AXRs [7].

## BARIUM ENEMA

Barium enema (BE) is a retrograde colonic assessment that can be done as either a single- or double-contrast study. A double-contrast BE—which is preferable except in cases of severe colitis—utilizes smaller amounts of high-density barium, followed by air insufflation. Double contrast ensures that the mucosa is coated with a thin layer of barium, while the lumen remains distended with air. It can be used to asses for active inflammation and to determine the extent of disease, but the role of BE has been reduced with the widespread availability of endoscopy. Barium enemas can also interrogate colonic strictures that preclude the passage of an endoscope. Information obtained in this setting includes stricture length, diameter and status of the colon proximal to the stricture.

**Figure 2.**
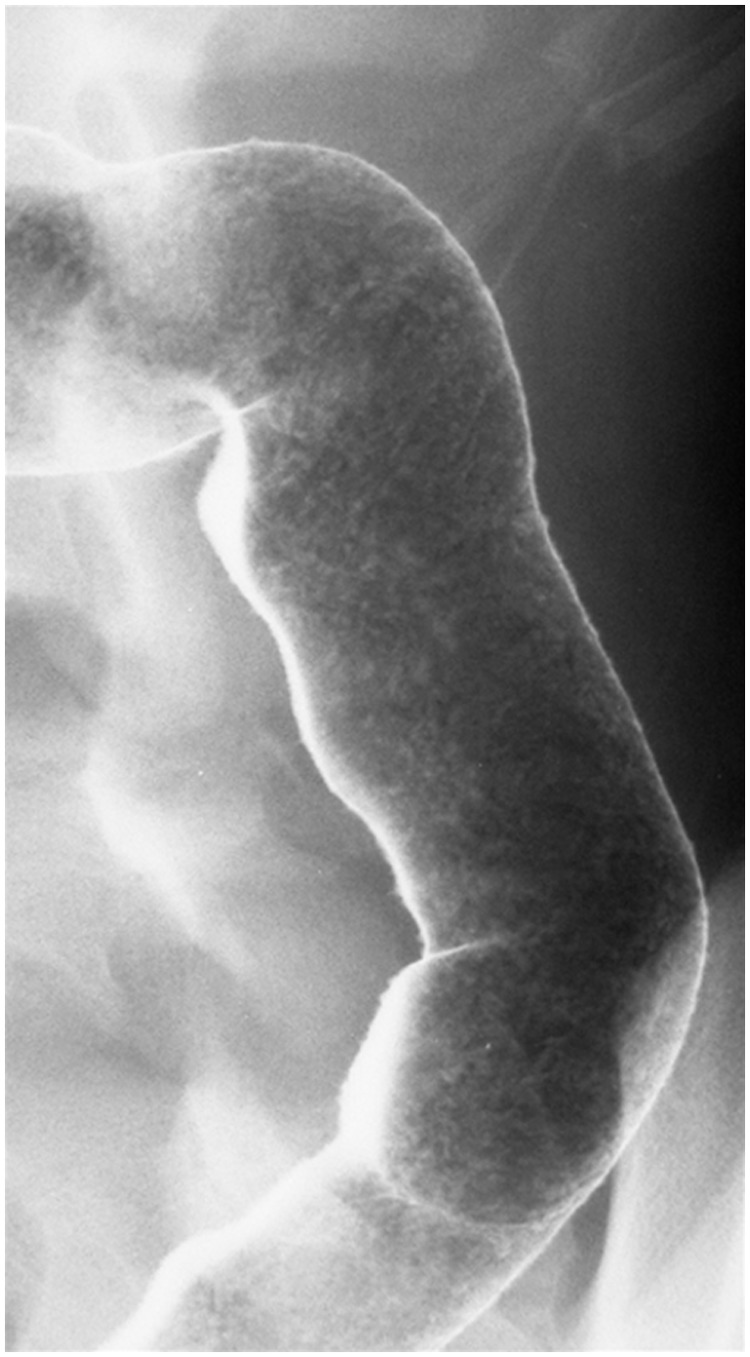
Double contrast barium enema illustrating granular mucosa in a patient with active ulcerative colitis. (Courtesy of Stephen W. Trenkner, MD).

Multiple inflammatory features have been described in the literature, including a fine mucosal granularity (Figure 2) caused by edema and hyperemia [5]. Adherence of flecks of barium to superficial erosions or ulcers superimposed on a background of mucosal irregularity may give an appearance resembling the stippling of paint. Deeper ulcerations into the submucosa with lateral extension produce the appearance of collar-stud or collar-button ulcers [8]. With extension of the ulcers, intervening normal mucosa may protrude, to appear as polyps. Chronic inflammation may result in shortening and narrowing of the colon and it can lose its interhaustral folds, resulting in a featureless or tubular appearance. Other reported changes due to chronic inflammation include widening of the presacral (retrorectal) space as seen on a lateral film of the rectum. Backwash ileitis may be seen in 15–20% of patients with severe UC, and it is manifested by a fixed, patulous ileocecal valve with a fine, granular-appearing terminal ileum.

The diagnostic yield of BE has been compared with both ileocolonoscopy and CTC in IBD patients. In a prospective study by Loose *et al.*, colonoscopy and BE were performed in 22 individuals with UC [9]. There was a substantial underestimation of the extent of disease on BE in four patients (18%) and three patients (14%) with a normal BE had pancolitis on endoscopy. BE has also been assessed in 33 Crohn’s colitis patients undergoing CTC [10]. CTC identified abnormalities proximal to a stenotic region in nine cases (not visualized on BE) which required medical or surgical intervention.

## COMPUTED TOMOGRAPHY

The use of CT has rapidly expanded in IBD patients. Various techniques can be utilized including standard CT abdominal and pelvis, CT enterography (CTE) and CT colonography (CTC). Standard CT can be performed with or without intravenous contrast, depending on the clinical setting. In comparison, CTE involves ingestion of large-volume neutral oral contrast agent along with iodinated intravenous contrast, typically in the enteric phase (50 seconds after injection), to maximize the visualization of enhancing intestinal lesions and inflammation. Multiplanar images are reconstructed with high spatial resolution (slice thickness ≤3 mm). CTC requires a colonic cathartic preparation and colonic distension with air insufflation administered via a rectal tube. CTC is designed for colonic rather than small bowel assessments.

There are multiple potential applications for CT in UC patients. Standard CT of the abdomen is often ordered to exclude complications of IBD, such as perforation. CTE can be used to assess for small bowel disease in IBD-U patients, evaluate colonic disease activity and extent in both CD and UC cases, detect penetrating complications—and it can diagnose extra-intestinal IBD manifestations. CTC has also been utilized to assess colonic disease activity. Biomechanical computed tomography is a novel image-analysis method that can measure bone strength in combination with a CTE or CTC protocol. In a study of IBD patients (UC, *n* = 45), dual energy X-ray absorptiometry (DEXA) and CTE-generated bone mineral density (BMD) *T*-score values were highly correlated [2]. This technology could potentially eliminate the need for DEXA scans in IBD patients already undergoing a CTE or CTC.

**Figure 3.**
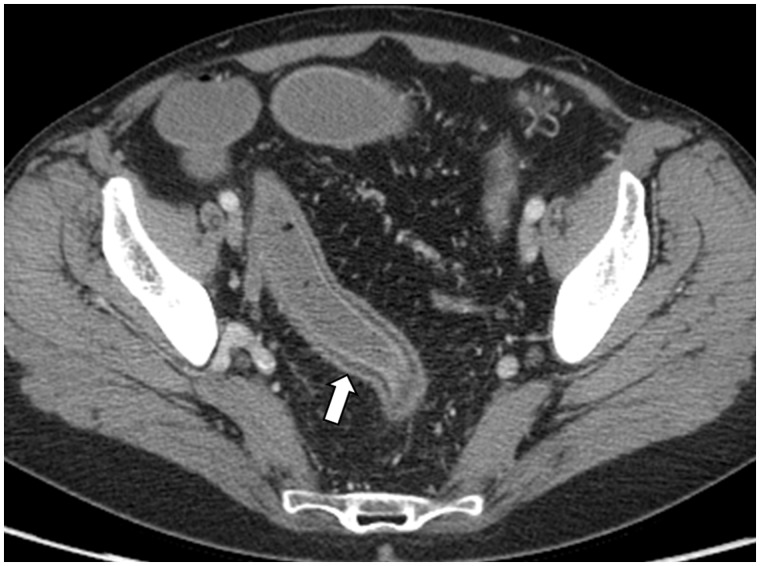
Computed tomography enterography in a 55-year-old female with pancolonic ulcerative colitis. White arrow highlights region with wall thickening and enhancement.

The radiological hallmark of active UC is the presence of colonic mural thickening and enhancement. Normal colonic wall diameter is in the range of 2–3 mm, whereas a mean wall thickness of 8 mm has been reported in UC patients with active disease (Figure 1b) [11, 12]. Approximately 70% of patients with active UC demonstrate inhomogeneous wall enhancement and stratification after intravenous contrast is administered (Figure 3) [11, 12]. Deposition of fat in the colonic wall is seen in up to 60% of UC patients. Radiological features of UC may also include rectal narrowing, widening of presacral space and stranding of perirectal fat [13].

There is a paucity of comparative CT data among studies of UC patients. As CTE was developed primarily for evaluation of Crohn’s disease, only a few studies have explored its use in UC. An overall CTE sensitivity of 93% and specificity of 91% has been reported for the detection of moderate-to-severe endoscopic inflammation in 35 IBD patients (UC: *n* = 20; CD, *n* = 15) with well-distended colons [14, 15]. The utility of CT (non-enterography protocol) in assessing the severity of UC was compared in 23 UC patients undergoing colonoscopy. Individual CT features (bowel wall thickening, hyperenhancement, mural stratification, mesenteric hyperemia, pericolonic stranding and enlarged lymph nodes), as well as the cumulative CT score (sum of inflammatory parameters), demonstrated statistically significant correlation with colonoscopic severity using the Mayo UC score (*P* < 0.0001); however, under CT examination, only intestinal wall thickening correlated with histopathological severity scores (*P* = 0.01) [16]. A small study of UC patients (*n* = 6) assessed using CTC demonstrated moderate correlation between loss of haustration, a rigid bowel wall and bowel thickness with UC endoscopic severity [17].

Overall, the limited data on CT assessments in UC support endoscopy as the standard of care for the assessment of disease activity, extent, and severity. CT is preferred to endoscopy in UC patients with impassable stenoses, comorbidities where colonoscopy is contra-indicated, or suspected disease complications such as perforation [18, 19].

## MAGNETIC RESONANCE IMAGING

Magnetic resonance imaging (MRI) is another non-invasive imaging option for UC patients. It can be performed as MRI of the abdomen and pelvis, MR enterography (MRE), MR colonography (MRC), or MR enterocolonography (MREC). For small bowel interrogations, large-volume enteric contrast agents can be administered orally (MRE) or via a nasojejunal tube (MR enteroclysis) [15]. Glucagon or other anti-peristaltic agents are often administered to alleviate motion artifact related to bowel peristalsis. MR colonography can be performed after a cathartic colon preparation has been given. Use of diffusion-weighted imaging with magnetic resonance colonography (DWI-MRC)—a technique which assesses changes in water diffusion—has been reported to assist with the detection of colonic inflammation in UC without requiring a bowel preparation [20]. Most MRC protocols do require colonic distension with water or contrast enemas except with DWI-MRC [21]. MRI imaging applications and indications in UC are similar to CT, except bone strength assessments, which have not been validated with MRE.

**Figure 4.**
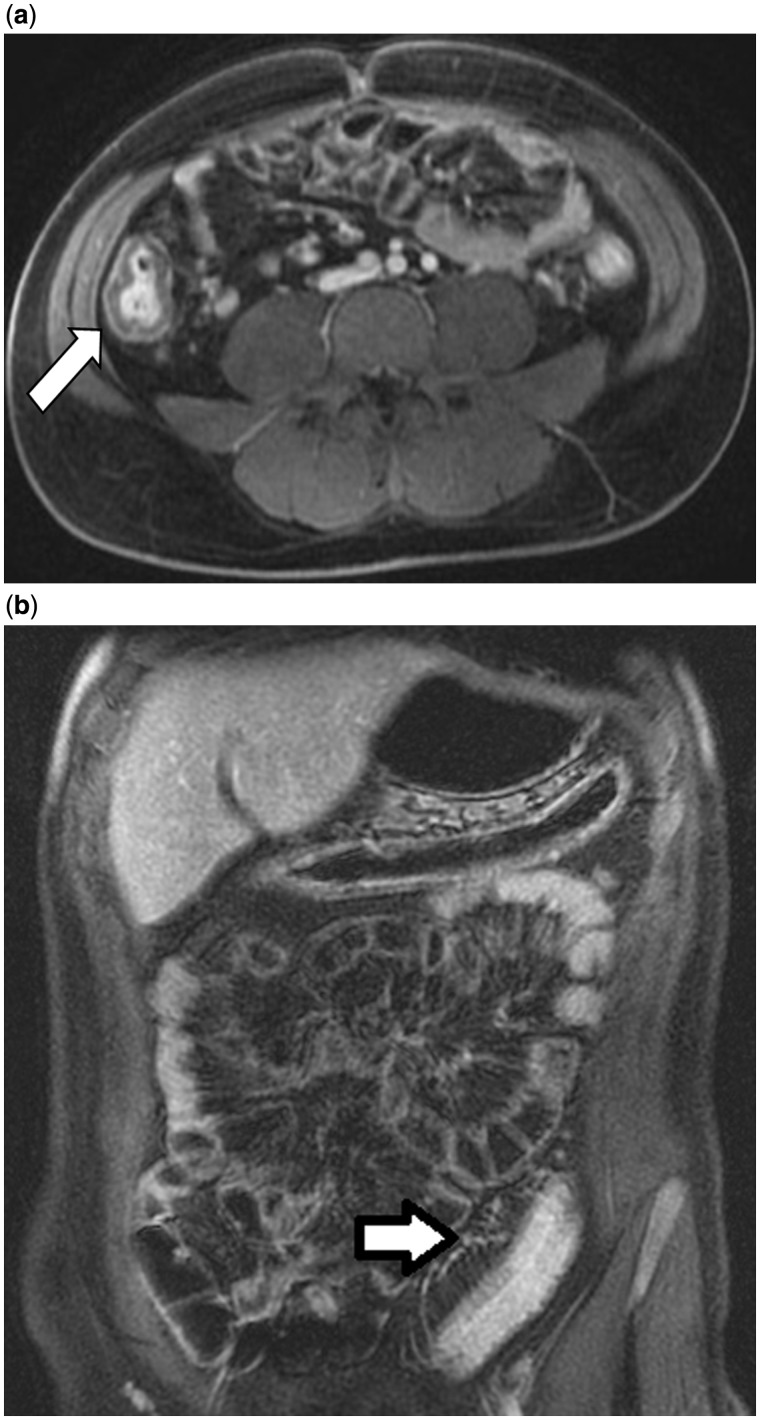
Magnetic resonance enterography in a 24-year-old male with ulcerative colitis. 4a: Colonic wall thickening (white arrow) and hyperenhancement of the right colon. 4b: Engorgement of the pericolonic vasa recta (white arrow) with colonic wall thickening and hyperenhancement in the sigmoid colon.

Multiple MRI findings for active intestinal inflammation have been reported in UC patients. This may vary depending on the MR technique, severity of inflammation and the chronicity of disease [22]. Mild active colonic disease may exhibit subtle thickening of the colonic wall and reduced distensibility. Moderate-to-severe disease may demonstrate thickening of the intestinal wall, mural edema, ulcerations, loss of haustra, mural hyperenhancement, engorged *vasa recta* (comb sign) and enlarged pericolonic lymph nodes (Figure 4) [22]. Increased disease chronicity has been described as resulting in loss of haustra, smooth contours, tubular narrowing, rigidity and fatty proliferation limited to a widened perirectal space [22].

MRI in UC patients has been assessed with colonoscopy as the reference standard. Sensitivities up to 88% and specificity up to 100% for active colonic inflammation have been reported for various MRC protocols [21, 23–30]. Less optimal performance was described in an MRC study by Schreyer and colleagues, in which patients received rectal contrast as a gadolinium enema [24]. Sensitivity on a per-segment analysis for active colonic inflammation in UC patients was only 58.8%. By comparison, utilizing a water enema for colonic distension, Ajaj *et al.* reported sensitivity and specificity values of 87% and 100%, respectively, for active colonic inflammation in 16 UC patients [25]. The largest MRC-based study in UC patients involved 50 patients receiving water enemas [21]. Independent predictors for endoscopic activity on a segment basis were relative contrast enhancement (RCE), presence of bowel wall edema, enlarged lymph nodes and the presence of engorged peri-enteric vasculature (comb sign). An MRC severity (MRC-S) index was reported as 0.125 × RCE (%) + 0.17 × edema + 0.19 × lymph nodes + 0.47 × comb sign [21]. MRC-S index ≥1 detected any endoscopic inflammation with a sensitivity of 87% and a specificity of 88%.

## ULTRASOUND

Ultrasonography (US) is a relatively new modality as applied to intestinal imaging. Oral bowel preparation is typically not required or utilized before a routine abdominal US [31]. Fasting six hours prior to the examination reduces air in the bowel that may hamper the examination. Oral administration of agents such as polyethylene glycol may improve image quality and diagnostic accuracy [32]. Intravenous contrast is utilized in some specialized centers, but it is not FDA approved for intestinal assessments in the United States. The technique of colon hydrosonography has been described and involves instillation of water into the rectum [32]. US for IBD often requires high-frequency (5–17 MHz) linear array probes to increase spatial resolution and to allow adequate assessment of the intestinal wall [33]. Doppler interrogations provide additional vascular information and may assist with differentiating predominantly inflammatory from fibrostenotic lesions.

Several potential indications for ultrasound exist for patients with ulcerative colitis. It has the advantages of avoiding exposure to ionizing radiation, it is low cost and it is well tolerated by patients. The ileocecal region, sigmoid-, ascending- and descending colons are adequately visualized in most patients, whereas visualizing the entire transverse colon can be challenging because of its variable anatomy and position. The rectum can be difficult to evaluate due to its position in the pelvis [33]. US has been utilized to define the anatomical location and severity of UC in patients with established disease and to assess for penetrating complications [34]. In addition to assessing for colonic inflammation, US can interrogate the small bowel to assess for Crohn’s disease in patients with IBD-U. It In a small study of 26 consecutive patients with steroid refractory or dependent UC, who were treated with granulocyte and monocyte adsorption apheresis, increased bowel wall vascularity on contrast-enhanced US correlated with clinically assessed non-response to cytapheresis [35]. Finally, endoscopic ultrasonography (utilizing a catheter probe) has been explored in terms of predicting response to medical therapy in severe UC cases [36].

Multiple features of active colonic inflammation have been described, based on US images. This includes wall thickening (>3 mm), complete or relative preservation of the echo-stratification of the colonic wall (except in severe disease) and loss of the haustra coli profile [32, 34]. The presence of thickened bowel wall in the terminal ileum—along with the presence of concomitant lesions such as strictures, fistulas or abscesses imaged with US—is suggestive of Crohn’s disease. Additionally, creeping fat and enlarged lymph nodes are less frequently described with UC, compared with CD [34].

The imaging performance of ultrasound in UC has been compared with other modalities. Overall, in four studies assessing the diagnostic accuracy of US in UC (*n* = 74), sensitivities ranged from 48–100% and specificities from 82–90% [37]. Evidence suggests that the diagnostic performance of US may be related to disease location, as sensitivity is high for sigmoid/descending colon disease (up to 98%), but low for rectal disease (15%) [34]. Other studies comparing US to endoscopy have reported an overall diagnostic accuracy of 89% in identifying active UC using a definition of bowel wall thickness >3 mm and an increased Doppler signal [27]. Studies have shown a strong correlation (*r* = 0.884) between the endoscopic activity index (EAI) and the US activity index (parameters include wall thickness ≥3 mm, stratification, increased Doppler signals, peritoneal surface thickening and loss of bowel compressibility) [27]. US has also been compared with MRI in a prospective study of 24 UC patients undergoing ileocolonoscopy, transabdominal US and MRI. Compared with MRI, US had a higher sensitivity (95 vs 67%) but comparable specificity (96 vs 95%) [27].

The concept of mucosal healing is an emerging treatment goal in IBD. The ability of US to assess responses to medical therapy was prospectively studied in UC patients with moderate-to-severe disease. Seventy-four patients, clinically responsive to corticosteroids, underwent follow-up colonoscopy and intestinal US at 3, 9 and 15 months. High concordance was demonstrated between endoscopic and US scores (weighted κ between 0.76 and 0.90). Additionally, a role for US in prognostication was also suggested by the study. Moderate-to-severe endoscopic and US scores at 3 months were associated with a high risk of endoscopic activity at 15 months (odds ratio (OR): 5.2; CI: 1.6–17.6) [38, 39].

## NUCLEAR MEDICINE IMAGING

### Scintigraphy

Scintigraphy is an imaging method that relies on radiotracers (radioactive isotopes) to detect active inflammation. Leukocyte scintigraphy utilizes physiological leukocyte migration to areas of active inflammation. Radio-labeling of the patient’s leukocytes (isolated from venous blood), using such agents as technetium-99 m hexamethyl propylene amine oxime (^99m^Tc-HMPAO) is required [40]. Another scintigraphic technique utilizes pentavalent ^99m^Tc dimercaptosuccinic acid (^99m^Tc-[V]-DMSA) with a suggested mechanism of inflammatory lesion localization due to infiltration of the interstitial space, caused by increased capillary permeability [41]. A gamma camera equipped with a collimator is utilized to scan the abdomen, with the patient in a supine position. This scanning has variously been carried out early (45 minutes) to late (3–4 hours) after injection of radiotracers [42, 43]. Colonic location is established by considering the large intestine as five areas, namely the cecum/ascending colon, transverse colon, descending colon, sigmoid and rectum [44]. Severity of colitis is graded, based on radiotracer uptake (on early and/or late scans) in comparison to bone marrow uptake (usually in the iliac crest) [44]. This is ranked as Grade 0 (no abnormal activity), Grade 1 (abnormal activity with an intensity less than bone marrow), Grade 2 (abnormal activity equal to bone marrow) or Grade 3 (activity more than bone marrow).

Scintigraphy can be used for the initial detection of inflammation in IBD, assessment of disease activity, extent of involvement, disease severity and treatment response [44, 45]. Advantages over endoscopy and other radiological imaging modalities include its non-invasive nature, the fact that it is well tolerated (no large-volume oral contrast is utilized) and it can visualize the entire gastro-intestinal tract [40]. WBC scintigraphy is also associated with relatively low radiation exposure (2–4 mSv per exam), making it well suited to the investigation of pediatric IBD when MRI, low dose CT, or US are not available [46]. Limitations of leukocyte scintigraphy include the high cost and time-consuming *in vitro* labeling procedure [40].

The role of leukocyte scintigraphy in UC diagnostic and management algorithms remains unclear. A study by Alberini *et al.* in 28 pediatric IBD patients (five individuals with UC) using ^99m^Tc-HMPAO-labeled white blood cells (^99m^Tc-HMPAO WBC) scintigraphy suggested that patients with negative scintigraphy scans may avoid unnecessary colonoscopy [47]; however Cucchiara and colleagues, after evaluating 21 pediatric IBD patients, concluded that whilst a positive test indicated the presence of inflammation, a negative result could not exclude cases with mild inflammation [48]. Another study compared the diagnostic performance of CT (non-enterography), ^99m^Tc-HMPAO WBC scintigraphy and colonoscopy in 313 consecutive pediatric patients (UC, *n* = 38). Scintigraphy had a high sensitivity (92%) and specificity (94%), however, CT was more likely to detect penetrating complications [49].

### Positron emission tomography

Positron emission tomography (PET) with 18-fluorodeoxyglucose (FDG) is a functional imaging modality which identifies inflammation, based on areas of increased glucose metabolism. Coupling CT or MRI to this adds anatomical data as well. Contrast-enhanced MRI may provide perfusion information that can be used in the pharmacokinetic modeling of PET data [50]. The appropriate role of PET has not yet been clearly established in UC. It has been suggested as a means of assessing extent of disease, disease activity and to measure response to treatment.

There is a paucity of data regarding the performance of PET imaging in UC patients compared with other modalities. In a study of 15 patients with mild-to-moderately active UC, modest correlation was demonstrated between PET/CT and colonoscopy for extent of disease (kappa 55.3%; *P* = 0.02) [51]. Another small study (*n* = 5) in patients with moderately active IBD (Crohn’s disease activity index (CDAI) ≥250 or an ulcerative colitis disease activity index >8), demonstrated significant reduction in inflammation on repeat PET/CT after initiation of medical therapy. The PET/CT response correlated with improvement in physician global assessment scores [52]. Rubin *et al.* studied the utility of FDG uptake in a cohort of 10 patients with well-characterized UC in endoscopic remission, utilizing PET/CT scans performed a mean of 37 days after a colonoscopy [53]. Three patients had elevated FDG uptake in the colon despite clinical remission and one patient with increased uptake in the terminal ileum was later found to have Crohn’s ileocolitis. Limitations of the PET scanning modality include cost and the ionizing radiation exposure [54].

## RADIOLOGICAL EVALUATION OF THE ILEO-ANAL POUCH

Restorative proctocolectomy with ileal pouch-anal anastomosis (IPAA) remains the surgical treatment of choice for the majority of patients with ulcerative colitis (UC) who fail medical therapy or develop dysplasia or neoplasia. The frequency of pouch failure has been estimated to be 6.8% during a median follow-up of 36.7 months and increases to 8.5% after more than 60 months [55]. The most common causes for early-onset pouch failure include anastomotic leaks, abscess formation and pelvic sepsis. Late-onset pouch failure etiologies include chronic pouchitis, CD of the pouch, refractory cuffitis, irritable pouch syndrome, pouch stricture, afferent loop syndrome and small bowel obstruction [56]. Given the considerable symptom overlap between the various inflammatory and non-inflammatory conditions, use of diagnostic imaging modalities in addition to pouch endoscopy (PES) is essential in order to distinguish between the etiologies and to identify extraluminal complications [57].

**Figure 5.**
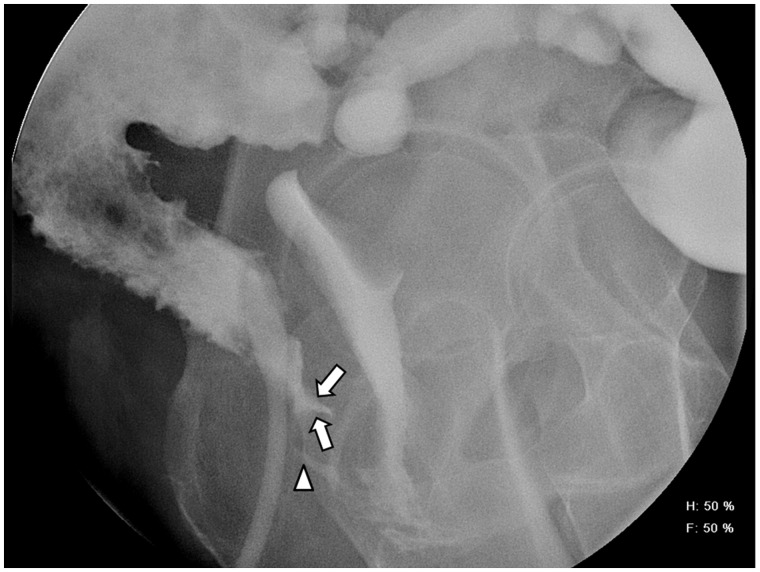
Pouchogram in a 49-year old female who presented with feculent vaginal discharge, with a prior history of ileal pouch-anal anastomosis surgery for UC. Sinus Tract (arrow) arising from the superior aspect of the anus, extending anteriorly just below the level of the ileo-anal anastomosis. Approximately 1 cm inferior to the sinus tract is a fistula (arrowhead) which extends to the mid-vagina and results in filling of the vaginal vault.

Various imaging options for acute and chronic causes of pouch failure include pouchogram, defecating pouchogram, scintigraphic pouch-emptying studies, pelvic MRI, CTE, MRE and defecating/dynamic MRI. Detection of leaks resulting in pelvic abscesses is often performed with a pouchogram, pelvic MRI, or CT scan [56]. Use of a CT scan has the additional benefit of guiding drainage of associated pelvic abscess [58, 59]. Difficulties with pouch evacuation can be further assessed with a pouchogram (structural abnormalities), defecating pouchogram, scintigraphic pouch emptying studies, defecating/dynamic MRI, and anorectal manometry with balloon expulsion [60]. Anorectal manometry is also useful for evaluating anal sphincter pressures in patients with fecal incontinence, especially once active pouchitis/cuffitis has been excluded with PES. Additional assessment of the sphincter complex using endoscopic ultrasound may be helpful in this setting as well [60]. A pouch sinus tract is usually a late presentation of an initial anastomotic leak, which can be detected using a combination of PES, pouchogram, MRI of the pelvis and examination under anesthesia (EUA) [56]. Pouch fistulae can arise as either a surgical complication or Crohn’s disease of the pouch (Figure 5). Fistula location and characteristics can be revealed using a combination of PES, pelvic MRI, pouchogram and EUA, with some studies suggesting superior accuracy from EUA, compared with other modalities in this setting [56]. CT and MRI inflammatory pouch parameters include wall thickening, peripouch adenopathy and mucosal hyperenhancement [61].

Limited comparative data exist for various imaging modalities in UC patients with IPAAs. A retrospective study by Tang and colleagues explored the benefit of pouchogram (gastrograffin enema), CTE, abdominal/pelvic MRI and PES in 66 patients, including 60 with an IPAA J-pouch and 50 individuals with a history of UC. Overall, the highest diagnostic accuracy for small bowel and inlet strictures was obtained from PES, while a similar accuracy was reported for MRI and PES in the diagnosis of outlet strictures (92%). Pouchogram was the most accurate tool for the diagnosis of fistula (84.8%) and sinus tracts (93.9%). The use of two imaging tests in combination increased accuracy to 100% for strictures, fistulas, sinus and pouch leaks [62].

## CONCLUSIONS

The role of radiological imaging in UC disease assessments is an expanding field. Colonoscopy remains the ‘gold standard’ for assessments of disease extent, activity, severity and for obtaining tissue. Imaging, however, can be a non-invasive alternative when tissue acquisition is not required, or it can complement endoscopic assessment. A large number of options are available to clinicians, with modality selection being driving by both patient and test-specific characteristic. The use of radiological assessments in UC will probably continue to expand in both diagnostic and management algorithms.

**Funding:** D.H.B has received research support from Given imaging.

**Conflict of interest:** D.H.B has served as a consultant for BRACCO and P.D has no conflicts of interest.
